# Women’s lifetime reproductive profiles and frailty among aging individuals in the USA and the Philippines

**DOI:** 10.1186/s40101-022-00300-5

**Published:** 2022-07-01

**Authors:** Michelle Escasa-Dorne, Ronza Zoorob

**Affiliations:** 1 Department of Anthropology, University of Colorado Colorado Springs, 1420 Austin Bluffs Parkway, Colorado Springs, CO 80918 USA; 2 Lake Erie College of Osteopathic Medicine, 1858 West Grandview Boulevard, Erie, PA 16509-1025 USA

**Keywords:** Aging, Frailty, Senescence, Philippines, USA, Cross-cultural, Life history theory, Reproduction

## Abstract

Frailty, particularly traits which are related to sex steroid hormone production, results from the age-associated decline in function considered to be part of the typical aging process. This process may vary with influences including environmental, biological, or lifestyle factors. An area of concern that has received relatively little attention is the impact of cumulative lifetime changes in sex steroid hormones related to the traits we see that typify the human aging process. Women’s lifetime reproductive profiles include menstrual/ovulatory cycles, gestation, and lactation, all of which respond to changes in sex steroid hormone levels. Here, we review data on reproductive profiles and risks of frailty among post-menopausal women. In the current study, our team collected reproductive histories of women to determine the estimated number of lifetime reproductive cycles (ELC). We interviewed 44 women in the USA and 67 women in the Philippines aged 65 years plus to obtain data on reproductive cycles, pregnancy, and breastfeeding. Participants completed several frailty tasks including grip strength, a sit-to-stand test, and gait speed. While ELC was not associated with frailty in the US population, higher ELC was associated with lower frailty among the Philippine population. The current study should be considered as an exploratory project investigating field-friendly methods to use when considering lifetime reproductive histories and the influence on the aging process.

## Introduction

The purpose of the current project is to determine life history tradeoffs between energy used for the purposes of reproduction versus maintenance among aging individuals in the Philippines and the USA. Women’s reproductive profiles include the start/stop of menstrual/ovulatory cycles, gestation, and lactation, all of which respond to changes in sex steroid hormone levels. Frailty, particularly traits which are related to sex steroid hormone production (e.g., reductions in bone density), results from the age-associated decline in function considered to be part of the typical aging process. An area of concern that has received relatively little attention is the impact of cumulative lifetime changes in sex steroid hormones related to the traits we see that typify the human aging process. Moreover, the ebbs and flows of sex steroid hormones of a human reproductive profile that includes a lengthy gestation and lactation time may not be as dramatic as the cumulative ebbs and flows of the sex steroid hormones that are present during the menstrual cycle (see [24] for an in-depth discussion on the reproductive hormonal profile of women).

### Literature review

Life history theory (LHT) is a widely used modeling structure for understanding human behavioral and physiological variation [6, 21, 25]. Life history theory suggests all organisms utilize a finite amount of energy towards three main areas of livelihood: growth, maintenance, and reproduction. Because resources are not unlimited, organisms must devote energy towards the three areas by determining the most optimal strategies. One of the implications of LHT is that energy devoted towards reproduction is energy that cannot be used for the other two categories, growth and maintenance. As such, it is hypothesized that individuals who invest more energy resources into reproduction may see a trade-off in maintenance of the body. Yet, previous data indicate significant variation in the health of aging individuals who have invested in multiple offspring. Some studies suggest there is a tradeoff with health later in life, a model referred to as antagonistic pleiotropy [14, 27], however, other studies suggest having more children, and breastfeeding for longer durations, may have a protective effect on certain aspects of health (e.g., reproductive cancers or stress levels; [9, 10, 15].

“Frailty” is a term often used to capture the deleterious or senescent properties of aging (for an overview of aging and frailty, see Crews [3]. While frailty does not appear to have a consensus definition in the literature reviewed (see [22]), it is often used as an overarching term to describe declines in function that can include motor, social, and cognitive factors. While the definitions appear to vary in the types of characteristics or traits included, here we use frailty to imply a sense of vulnerability, an inability to maintain bodily homeostasis, and/or a decline in health. Here we are interested in one primary area of frailty, reduced physicality, i.e., declines in strength, speed, and mobility.

Estrogens have been implicated in numerous areas of health that are often associated with the aging process. For example, elevated estrogen levels are associated with atherosclerosis [13, 20]. Men with higher than average estrogen levels have a higher risk of strokes and coronary artery disease [1, 5, 28]. Estrogens play an important role in the formation and function of bone cells, with low estrogen levels during the aging process implicated in bone density disorders such as osteoporosis and an increased risk of bone fractures in both women [12, 24] and men [16, 18].

The antagonistic pleiotropy hypothesis of aging argues that a decline in health with age is a result, or byproduct, of selection for the most beneficial health during the reproductive years [14, 27]. Some arguments in favor of this model suggest that an increase in reproductive health in the early years may pose a negative effect in later life. One example which has gained recent attention is the BRCA1 gene, which provides a benefit during the early reproductive years by increasing estrogen production and increasing reproductive viability, but correlates with increased breast cancer risk in later life [23].

Estrogens are an informative proxy for measuring tradeoffs between reproduction and later health, as it plays an important role in several areas of health. Certain life events see changes in estradiol levels, such as breastfeeding and pregnancy. For example, estradiol (the main form of estrogen) is crucial in the ovulatory cycle and for maintaining pregnancy; however, lifetime exposure to higher estradiol levels has been associated with an increased risk in certain types of breast cancers and osteoporosis [24]. While osteoporosis is often linked to a decrease in estradiol, one hypothesis suggests that it may be a sudden drop in estradiol, or repeated changes in estradiol, which may be a factor in the risk of osteoporosis after menopause [24]. While bone density can provide information on the present bone health of individuals (and thus, body maintenance towards bone structure, it also can be used as one measure of lifetime estrogen exposure (Forsmo 2001 [8], Nguyen 2000 [17], one variable which is receiving growing attention for its association with reproductive cancers in later life. In line with the antagonistic pleiotropy hypothesis, an increase in estradiol may be beneficial for increasing reproduction in earlier years, while posing a negative impact on health in later years.

These questions are relatively novel to ask, particularly with the framework of lifetime effects of reproductive profiles on the aging process. However, research into frailty and the numerous factors that may influence frailty tend to focus on US and Western populations. Here, we look at two populations: the USA and the Philippines. The Philippines, like many countries, has a growing number of aging individuals in their population, with the older adult population (age 60 and older) increasing by over 35% over the last two decades (Help Age Global Network, 2017 [11]). The Philippines tends to have a higher fertility rate of 3.1 children per woman compared to the USA which has around 1.7 children per woman (Help Age Global Network, 2017 [11]). Many Filipinos see both positives and negatives in the aging process. For example, Valdez et al. [26] reported that Filipinos seem to have more social support, better perspectives based on life experiences, and a positive outlook regarding productivity and experiences. However, this same study found that older Filipinos also report negative perspectives of aging that include physical decline. Still this study found that Filipinos appeared to view the aging process more positively than negatively. A study by Badana and Andel (2018) reported that key issues regarding aging involved social welfare, a fear of having to rely financially on their children or younger family members, and the burdens of health care. In our interviews with Filipino participants, we heard frequently a statement of “bata pa ako” (I am still young) said in a joking manner when describing to participants that we were conducting a study of aging. This seems to echo what other studies have found of aging populations in the Philippines [7]. Other studies seem to find a common ideology of “resilience” or “responsibility” as one of the traits of aging among Filipinos (see [2, 4, 26]). The biological and cultural intertwining of the aging process marks this, and studies of a similar perspective, as an important anthropological insight into variations in the aging process.

## Methods

Participants over the age of 65 were recruited to complete a questionnaire and short interview collecting data on the participants’ reproductive history, including variables relevant to estimating the number of lifetime menstrual cycles (e.g., menarche, number of children, time spent exclusively and non-exclusively breastfeeding, birth spacing, and menopause). Participants also completed the Positive and Negative Affect Schedule (PANAS) survey and the Duke Social Support Index (DSSI) surveys to assess for overall well-being, although we also screened participants for any ailments that would impact their ability to participate fully in the study (e.g., we excluded participants if they had a diagnosis of dementia or Alzheimer’s disease, as a large part of our study relied on memory recall). Table 1 provides an overview of participant demographic information.

**Table 1:** Demographic information of the participants

Demographics	US sample (UCCS Aging Center; *n* = 44)	Philippine sample (Manila, Baler; *n* = 67)
Age	65–90 (*m* = 69.36, SD = 5.2)	64–87 (*m* = 73.05, SD = 6.2)
BMI	25.4 (SD = 5.6)	22.6 (SD = 4.6)
Education	High school = 13.3% Associates/bachelors = 40% Masters/doctorate = 44.5%	High school = 13.2% Associates/bachelors = 31.6% Masters/doctorate = 2.6%
Age at menarche	13 (SD = 1.3)*	14 (SD = 2.3)*
Age at menopause	47 (SD = 8.5)*	49 (SD = 4.5)*
Parity	1.5 (SD = 1.3)*	3.5 (SD = 2.5)*
Months breastfeeding	8.7 (SD = 18.6)*	20.9 (SD = 42.5)*
Estimated lifetime cycles	398	390

### US sample—UCCS Aging Center

Participants were recruited from the University of Colorado Colorado Springs (UCCS) Research Participant Registry list, which is a list of potential participants who have agreed to be contacted for studies conducted through or at UCCS. The registry requires pre-approval through the University’s IRB process. We requested access to the list of participants over the age of 65. After receiving the participant registry access, we contacted potential participants. Participants were interviewed at the UCCS Lane Center for Academic Health Sciences.

### Philippine sample—Manila and Baler

Participants were recruited from two locations in the Philippines. These locations were selected to allow us to recruit participants from a larger, metropolitan city and a smaller, less urban location. These locations were also selected due to local contacts in these areas. One location was in the municipality of Baler, the capital province of Aurora. Baler is located on the northeast region of Luzon, the largest and most northern island of the Philippine Islands. Participants were recruited through word of mouth, and we conducted home visits for each participant. In many cases, multiple participants lived in the same home or neighboring homes, allowing us to collect multiple participants in one setting.

Participants were also recruited from Paranaque, a city that is part of the larger Metropolitan Manila (“Metro Manila”) capital region. In Paranaque, we recruited through word of mouth at two main barangays (“neighborhoods”) at the barangay hall. We completed the methods either at the barangay hall or at a nearby grade school.

Prior to the start of the study, we translated the interview questions into Tagalog with local speakers of the language. Participants were provided with the option to complete the study in English or Tagalog. If participants opted to complete the study in English, we were present and available if any translations or explanations were needed in Tagalog.

### Measures of frailty

After completing the interview and questionnaire, we measured each individual’s grip strength, gait speed, and a sit-to-stand count. We use grip strength as an indicator of strength, gait speed as an indicator of speed, and sit-to-stand count as an indicator of mobility. We selected these particular measures as they are field-friendly methods due to ease of transport and portability. All methods were reviewed by the University of Colorado Colorado Springs Office of Sponsored Programs and Research Integrity and approved under IRB Protocol 17–039 (US study) and 18–046 (Philippine study).

#### Grip strength

Participants used a dynamometer using the non-dominant arm with their elbow at a 90-degree angle and with the elbow/upper arm not touching the upper body for between 1 and 3 s. Participants repeated this two times, resting in between, and the average of the two was used for the remaining analyses.

#### Gait speed

In a majority of cases, we aimed to have the same distance walked between participants. This was simple to accomplish at the Colorado location, but less consistent in the Philippine locations. As much as possible, we measured the amount of time participants walked 10 m. We measured a 2-m section to be the “acceleration zone,” which accounts for the speed required for the participant to gain for full stride. We started timing at the end of the 2-m mark and continued timing until the participant reached between 4 and 10 m (depending on the feasibility of the testing location) in length. The last 2 m are the “deceleration zone” which were not timed (see Table 1). We asked the participants to walk the full length at a pace that is comfortable but not competitive (e.g., about the pace they might use to cross a street or walk in the mall). In the final calculations, we include the space walked (not including the acceleration and deceleration zones) to calculate gait speed per meter

#### Sit to stand

We measured the number of times the participant was able to move from a seated position to a standing position within a 30-s time frame. Participants sat in a solid, non-rolling chair with their feet firmly on the ground, and about shoulder-width apart. Participants sat in the middle of the chair, or such that the feet remained planted on the ground, and with their hands crossed over the chest so that the participants’ back was not braced nor were they able to use the chair to assist in lifting the participant up and out of the chair (see Table 2). The participant completed as many sit-to-stand motions as comfortably as possible, without exerting effort to the point of exhaustion.

**Table 2:** Data for the three measures of frailty

	**USA**	**Philippines**
Grip strength (pounds pressure)	20.69 (SD = 5.4)	12.09 (SD = 4.9)
Gait speed (seconds)	4.44 (SD = 8.2)	1.36 (SD = 4.2)
Sit to stand (repetitions)	11.05 (SD = 3.2)	9.12 (SD = 0.4)

### Estimated number of lifetime cycles

With the reproductive histories collected from the questionnaire, we calculated the estimated number of lifetime menstrual cycles following a formula derived from Fox and colleagues (2013). This was done by taking the number of years cycled (menarche to menopause) and assuming an average of 1 cycle per month. From this number, we subtracted the number of months spent pregnant, assuming 9 months per pregnancy, and months spent exclusively breastfeeding. We then subtracted 50% of the months spent partially breastfeeding. This number was subsequently denoted as an individual’s estimated lifetime cycles (ELC).

All data were entered into a spreadsheet and analyzed using SPSS v. 23. Unless otherwise specified, all data were treated as two-tailed with *α* = 0.05. All data were tested for normality prior to further standardized statistical testing. Group differences were assessed through parametric *t*-tests and ANOVAs. Effects of correlations were analyzed through Pearson’s (or Spearman’s rho) correlational tests.

## Results

Forty-four women from the UCCS Aging Center and 67 women from the Baler and Manila regions participated in the study. The ages of the participants ranged from 65 to 90 years of age (UCCS *m* = 69.36, SD = 5.2; Philippines *m* = 73.05, SD = 6.2). Demographic information on the participants can be found in Table 1. Samples were significantly different in age at menarche (*F* = 15.80, *p* < 0.001), age at menopause (*F* = 6.44, *p* = 0.01), parity (*F* = 9.60, *p* = 0.003), and months exclusively breastfeeding (*F* = 19.95, *p* < 0.001) though not in ELC (*F* = 0.49, *p* = 0.49).

Estimated number of lifetime cycles (ELC) was not correlated with our measures of frailty (see Table 2 for averages of the frailty tasks for each sample). ELC was not correlated with grip strength (*F* = 0.91, *p* = 0.625), gait speed (*F* = 1.49, *p* = 0.351), nor number of completed sit-to-stand cycles (*F* = 1.2, *p* = 0.464). ELC was also not correlated with reported overall health (PANAS) (*F* = 0.63, *p* = 0.817) nor overall mental wellness (DSSI) (*F* = 1.15, *p* = 0.489). However, ELC was correlated with BMI (*F* = 6.42, *p* = 0.023).

Using ELC, we grouped participants into upper and lower cycle groups using the mean ELC (*m* = 398). We then used high versus low lifetime cycles to analyze variations between the two groups and measures of frailty. These two groups were also not significantly different from one another in grip strength (*t* =  − 0.10, *p* = 0.921), gait speed (*t* =  − 0.57, *p* = 0.570), nor the number of completed sit-to-stand cycles (*t* = 1.97, *p* = 0.291) among the US sample. Using the Philippine sample, high cycle versus low cycle groups did not differ in grip strength (*F* = 1.81, *p* = 0.32), nor gait speed (*F* = 0.03, *p* = 0.57). There was a trend towards cycle group differences in the sit-to-stand task (*F* = 0.54, *p* = 0.07) with individuals who had more cycles completing more repetitions of the sit-to-stand task.

## Discussion

Our study sought to test whether lifetime reproductive cycles, as reported during interviews with women regarding their reproductive histories, impact some of the estrogen-related traits of frailty and aging, as measured through grip strength, mobility, and gait speed. The estimated number of lifetime menstrual cycles (ELC) was measured by taking the number of years menstruating (menopause − menarche), subtracting the number of total months pregnant, the number of months exclusively breastfeeding, and 50% of the months partially breastfeeding. This is a method that appears to be feasible in interview settings and can be used in field sites with ease.

ELC was not correlated with grip strength, sit-to-stand, nor gait speed. Two groups were formed into “high cycles” and “low cycles” using the median number of cycles of ELC. These two groups also did not differ from one another in ELC. While ELC was not associated with frailty in the US sample, higher ELC trended toward lower frailty among the Philippine sample. We do not know why this may be the case in the Philippine sample while not in the US sample. Further research should include additional measures likely relevant to understanding how lifetime reproductive traits may influence the aging process. Such measures might include additional assessments of factors contributing to frailty and ideally more specific biomarkers of sex steroid levels. Other indices which could be helpful and may influence lifetime sex steroid levels include activity levels, certain health parameters, or exogenous exposure to sex steroid hormones.

## Limitations and conclusion

The current study is meant to be an exploratory initial investigation into the effects of lifetime reproductive traits on the traits of the aging process. This study is not meant to be an extensive study of the traits we see during the typical aging process; however, this study aims to further understand how the accumulation of traits influencing the sex steroid levels over the lifetime influence the traits that sex steroid levels affect during the aging process. Specifically, this study aims to investigate one aspect of lifetime reproductive traits (number of menstrual cycles, caregiving/parenting) and one aspect of the aging process, associated with frailty. This study does not propose to review the numerous other traits that may be influenced by sex steroid levels during aging (e.g., a decline in skin elasticity, loss of libido, an increased risk of reproductive cancers, and memory loss), all of which may also be influenced by the accumulation of sex steroid levels (see [12]). In our current study, we did not include the use of hormonal contraceptives in the calculation of lifetime reproductive traits. The use of hormonal contraceptives would certainly impact an individual’s sex steroid hormone levels and could further be included in a calculation of lifetime sex steroid exposure. The use of hormonal contraceptives is relatively low in the Philippines, partially due to the difficulty to access these forms of medicine. While currently, 54% of married women in the Philippines report using a family planning method, in 1993, only 25% of women reported using family planning methods [19]. Furthermore, of the 54% of married women who report using a family planning method, approximately 27% of those used a hormone-based method (pill, implant, or injectable) while the rest reported the use of non-hormonal methods (e.g., traditional methods such as withdrawal or calendar tracking) [19].

Frailty measures are a field-friendly method of collecting data to analyze the trade-offs between reproduction and maintenance among aging populations. The measures used in the current study were easy to adapt to various scenarios in the USA and the Philippines.

Estimated lifetime reproductive cycles (ELC) are a useful tool when considering lifetime exposure to sex steroid hormones. However, ELC is limited by participants’ accurate recollection of their reproductive histories. There may be numerous factors that influence lifetime exposure to sex steroid hormones that are not captured by the current method of calculation, for example, hormonal birth control use, environmental exposure to sex steroid hormones, and/or medications. Moreover, frailty may be influenced by factors not captured by the current methodology, for example, activity patterns (including type of labor, physical limitations, etc.).

While there are certainly challenges that are posed by field-based research studies, including the current study, certain methods may be employed to assist with these difficulties. Here we used several methods that are field-friendly including portable equipment such as a dynamometer and calibrating different walking paths based on the space available to calculate walking speed. Additionally, using interviews to capture reproductive histories appear to be a minimally invasive method for investigating lifetime reproductive profiles. The interviews also provide the added means of identifying crucial sociocultural and interindividual variation. The current exploratory project may provide data sufficient for future studies investigating lifetime profiles of reproduction and trade-offs with other areas of life history.

## Data Availability

The datasets used and/or analyzed during the current study are available from the corresponding author on reasonable request.
